# Author Correction: Deep reinforcement learning for active flow control in a turbulent separation bubble

**DOI:** 10.1038/s41467-025-57534-x

**Published:** 2025-04-24

**Authors:** Bernat Font, Francisco Alcántara-Ávila, Jean Rabault, Ricardo Vinuesa, Oriol Lehmkuhl

**Affiliations:** 1 Faculty of Mechanical Engineering, Delft University of Technology, Delft, Netherlands; 2 Barcelona Supercomputing Center, Barcelona, Spain; 3 FLOW, Engineering Mechanics, KTH Royal Institute of Technology, Stockholm, Sweden; 4 Independent researcher, Oslo, Norway

**Keywords:** Mechanical engineering, Aerospace engineering, Applied mathematics, Fluid dynamics

Correction to: *Nature Communications* 10.1038/s41467-025-56408-6, published online 07 February 2024

In the version of the article initially published, the table in the lower half of Fig. 7 was missing and is now amended in the HTML and PDF versions of the article.

